# A Correlative Study of Interfacial Segregation in a Cu-Doped TiNiSn Thermoelectric half-Heusler Alloy

**DOI:** 10.1021/acsaelm.2c00699

**Published:** 2022-08-23

**Authors:** John E. Halpin, Benjamin Jenkins, Michael P. Moody, Robert W.H. Webster, Jan-Willem G. Bos, Paul A.J. Bagot, Donald A. MacLaren

**Affiliations:** † SUPA, School of Physics and Astronomy, University of Glasgow, Glasgow G12 8QQ, U.K.; ‡ Department of Materials, University of Oxford, Department of Materials, Parks Road, Oxford OX1 3PH, U.K.; ⊥ Institute of Chemical Sciences and Centre for Advanced Energy Storage and Recovery, School of Engineering and Physical Sciences, Heriot-Watt University, Edinburgh EH14 4AS, U.K.

**Keywords:** atom-probe tomography, analytical electron microscopy, grain boundary segregation, Heusler alloys, thermoelectrics

## Abstract

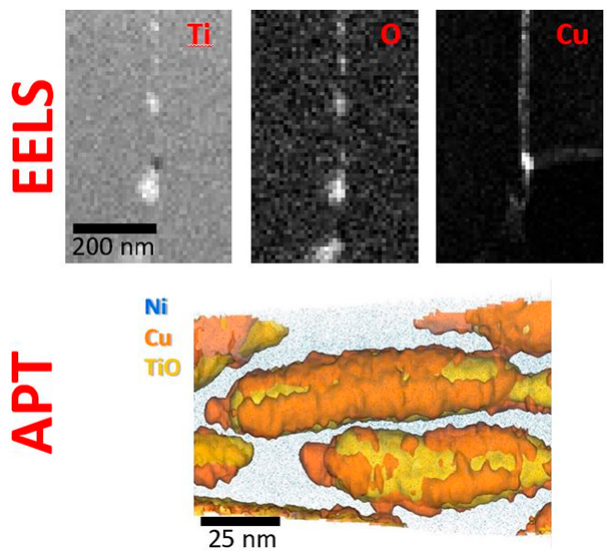

The performance of thermoelectric materials depends on both their atomic-scale chemistry and the nature of microstructural details such as grain boundaries and inclusions. Here, the elemental distribution throughout a TiNiCu_0.1_Sn thermoelectric material has been examined in a correlative study deploying atom-probe tomography (APT) and electron microscopies and spectroscopies. Elemental mapping and electron diffraction reveal two distinct types of grain boundary that are either topologically rough and meandering in profile or more regular and geometric. Transmission electron microscopy studies indicate that the Cu dopant segregates at both grain boundary types, attributed to extrusion from the bulk during hot-pressing. The geometric boundaries are found to have a degree of crystallographic coherence between neighboring grains; the rough boundaries are decorated with oxide impurity precipitates. APT was used to study the three-dimensional character of rough grain boundaries and reveals that Cu is present as discrete, elongated nanoprecipitates cosegregating alongside larger substoichiometric titanium oxide precipitates. Away from the grain boundary, the alloy microstructure is relatively homogeneous, and the atom-probe results suggest a statistical and uniform distribution of Cu with no evidence for segregation within grains. The extrusion suggests a solubility limit for Cu in the bulk material, with the potential to influence carrier and phonon transport properties across grain boundaries. These results underline the importance of fully understanding localized variations in chemistry that influence the functionality of materials, particularly at grain boundaries.

## Introduction

Thermoelectric (TE) generators are solid-state devices that convert waste heat directly into electricity. Extensive studies are driving their deployment as part of global activities for improved energy efficiency.^1,2^ Their wide-scale adoption is impeded by the high cost of scarce materials such as bismuth telluride and the low heat-to-electricity conversion efficiencies that are typical of more economic TE materials. Among a host of TEs, the intermetallic materials known as half-Heusler alloys (hHAs) are leading candidates for commercialization because they are based on abundant elements as well as being chemically and mechanically stable, and they can be designed with favorable intrinsic TE efficiency.^3^ This broad family of materials adopts a face-centered cubic (*F*4̅3*m*) structure with an XYZ-type composition and is particularly amenable to optimization through doping into “empty” crystallographic sites to simultaneously enhance the electronic carrier concentration and introduce phonon point scattering. These dual effects are important because the ideal TE material is a good electrical conductor and poor thermal conductor: it must produce usable electric current across a strong thermal gradient. However, unlike doping into elemental semiconductors such as Si and Ge, doping into hHAs can drive phase changes, segregation effects, and nanostructuring that will affect TE performance. Additionally, the polycrystalline nature of bulk TE materials introduces internal grain boundaries (GBs) that often have differing chemical compositions and tend to give rise to parasitic electrical resistances. Intriguingly, on the other hand, there is potential to “engineer” GBs as electron-transmitting, phonon-blocking layers that could mitigate against losses and also improve performance, such as a recent study of Te extrusion from Bi_0.5_Sb_1.5_Te_3_ during processing to induce favorable GB nanostructuring.^4^ Such grain boundary engineering has recently been discussed in a number of other thermoelectric systems, including Mg_3_Sb_2_,^5^ SnTe,^6^ SrTiO_3_,^7^ and Heusler alloys.^8−11^ In developing high-performance TEs—as with many polycrystalline functional materials—there is a need to characterize structural details that span multiple length-scales. Here, we outline a methodology derived from a variety of characterization techniques that provide structural insight ranging from micrometer phase segregation down to atomic-scale site occupancy.

Our focus is TiNiSn, in which we have previously shown Cu doping to produce an n-type hHA with enhanced phonon point scattering that usefully lowers lattice thermal conductivity.^12^ It is also inexpensive compared to other candidate TEs and supports commercially promising TE performance.^13^ It is vital to understand the distribution of Cu throughout the bulk material, to assess phase segregation effects that we have seen in other hHA systems and to determine the nature of GB structures. In previous reports, we have shown that synthesis via hot-pressing of elemental powders yields dense, polycrystalline TiNi_*x*_Cu_*y*_Sn materials. Bulk diffraction has indicated excellent agreement with the expected *F*4̅3*m* structure, although clustering and segregation effects of Cu at low dopant levels are difficult to discern through Rietveld refinement of fits to diffraction data. Bulk techniques including diffraction are also relatively insensitive to spatially localized defect structures, including GBs, that can affect performance. We previously used scanning transmission electron microscopy (STEM) to reveal the formation of Cu “wetting layers” that appear to facilitate the formation of coherent, low-angle GB structures.^13^ Here, we describe a second, rougher GB structure with a three-dimensional (3D) character that is hard to study with transmission electron microscopy (TEM) techniques alone. We instead used atom-probe tomography (APT), which offers near-atomic-scale characterization of microstructures in 3D and with full chemical resolution, to explore the microstructure of these rough GBs and the composition of the surrounding material. Metallic Cu is again found to segregate at the GB, alongside oxide precipitates and other impurities that can all be expected to affect both electronic and thermal transport. There is no obvious nanostructuring within the bulk material, and our results help to quantify the limit of Cu solubility in the bulk TiNiSn matrix. As the nature of grain boundaries is essential to the performance of a variety of advanced materials, from photovoltaics to structural materials, we anticipate the methodology of complementary, multilength-scale analyses outlined here to have broad applicability.

## Methodology

The TiNiCu_0.10_Sn samples were prepared by standard solid-state methods as part of a larger batch with different compositions. Metal powders of all four elements (Alfa Aesar, >99.99% purity) were mixed before being initially cold-pressed into 13 mm diameter pellets. These were then annealed under various controlled conditions before being finally hot-pressed; full details are provided in ref (13).

Following synthesis, samples were cleaved, mounted, and polished for scanning electron microscopy (SEM) examination and APT/TEM specimen preparation in a Thermo Scientific Helios G4 P-FIB UXe DualBeam FIB/SEM instrument (University of Glasgow). Use of such Xe plasma focused ion beam instruments as an alternative to Ga^+^ ion systems has been growing in recent times; they offer advantages such as significantly reduced sample beam damage, faster milling rates, and the possibility to produce novel APT specimen geometries without the need for lift-out methods.^14,15^ In the current work, a key goal was to correlate APT data through specific grain boundaries identified using EBSD. Samples were FIB-milled with a series of fiducial marks, and EDS analysis (Bruker Xflash) was performed during SEM to map the global chemistry across 100 μm length-scales. Elemental quantification was performed on a pixel-by-pixel basis by integrating the background-subtracted X-ray counts within the K_α_ peaks of Ti, Ni, and Cu and L_α_ peaks of Sn, with the summed signals collected across the entire field of view normalized to the composition ratio of the elemental powders used in synthesis. Electron backscattered diffraction (EBSD, Bruker eFlash FS) was used to map the surface granularity and to identify suitable grain boundaries, so that cross-sectional specimens for TEM analysis and needle specimens for APT studied could be produced from selected sites using standard lift-out methods.^16−18^

Scanning transmission electron microscopy (STEM) incorporating energy dispersive X-ray spectroscopy (EDS) and electron energy loss spectroscopy (EELS) was performed on a JEOL ARMcFEG instrument that was operated at 200 kV and was equipped with a Bruker XFlash EDS detector and a Gatan Quantum EELS spectrometer. APT specimens were analyzed on a Cameca LEAP 5000X HR (Oxford Materials). APT experiments were conducted at a stage temperature of 50 K, using both laser pulsing (100 pJ pulse energy, 355 nm ultraviolet laser) and voltage-pulsing (25% pulse fraction) modes. A detailed assessment of the differences between APT ionization mechanisms is provided elsewhere.^19^ Broadly speaking, the laser-pulsed mode is favored here for stoichiometric analysis, as it was found to yield more accurate results than voltage-pulsing and is more reliable when looking at inhomogeneous, oxide-decorated GBs, especially in these materials of relatively low thermal conductivity. Voltage-pulsing, in contrast, was preferable for crystallographic analysis of regions away from the GBs. We previously identified a trend for lower laser pulse energies returning more accurate chemistries in this material, but depending on the needle geometry (including the tip radius, needle length and diameter, support mounting, etc.), higher pulse energies did not always compromise the accuracy of results. The resulting data were reconstructed and analyzed using IVAS 3.8.8 visualization software (Cameca). Reconstructions were carried out by voltage-profile fitting, using an averaged evaporation field value for the three main alloying elements and ensuring all reconstructed densities were close (within ∼5%) to known material density.

## Results

### “Bulk” Analysis

**Figure 1:**
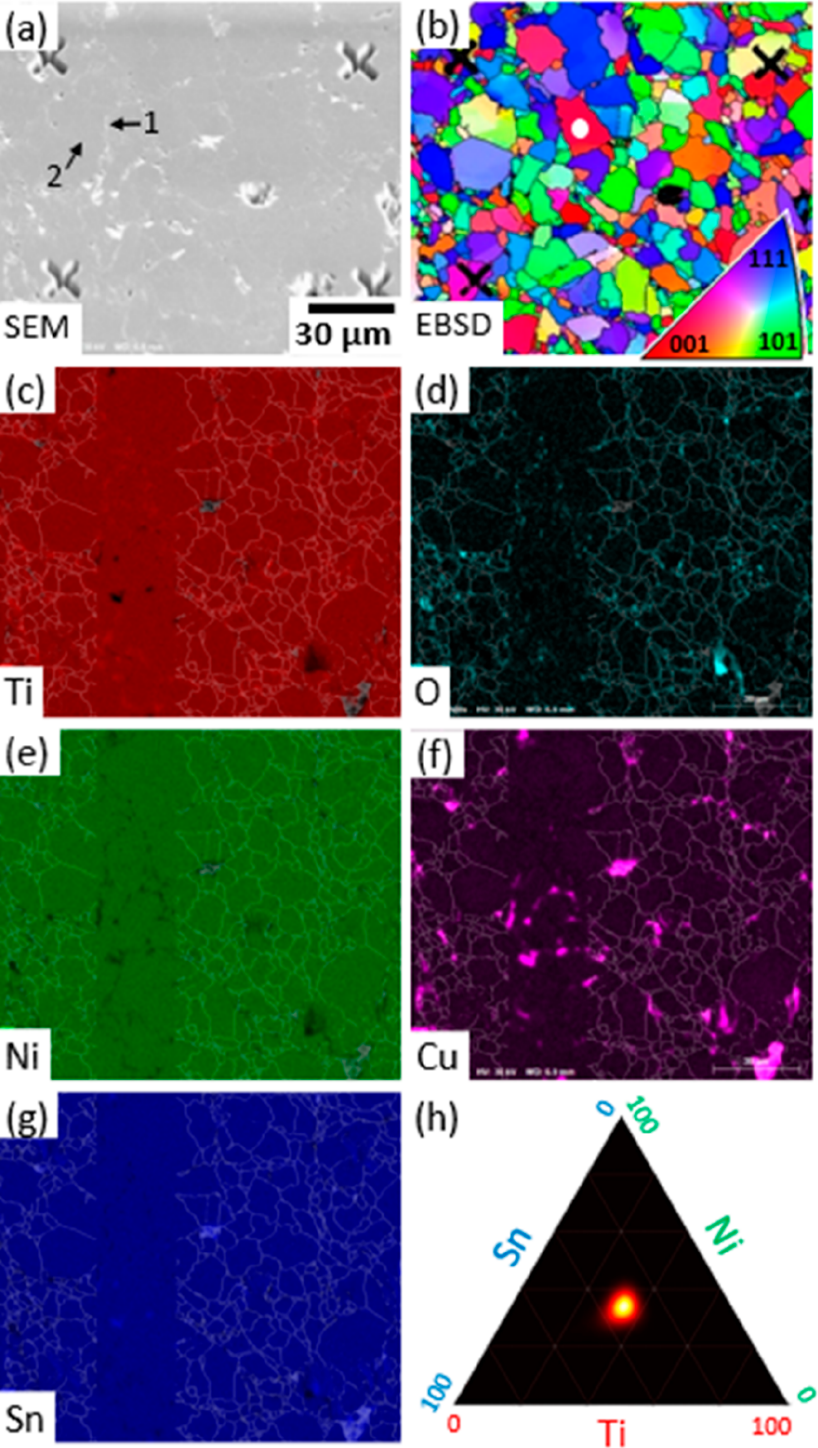
Electron-beam analysis of a polished TiNiCu_0.1_Sn surface. (a) An SEM image, with a scale-bar that relates to all subsequent images. The prominent cross features are reference markers milled into the sample using a focused ion beam. Arrow 1 indicates a “rough” grain boundary that is found to be decorated by oxides that appear white in the SEM image; arrow 2 indicates a more regular grain boundary that lacks oxides and so is harder to discern in SEM; see text for discussion. (b) An EBSD map of grains, with colors indicating the grain orientation (see key, inset). The white dot indicates the grain selected for analysis of “bulk” composition. (c–g) EDS elemental maps of Ti, O, Ni, Cu, and Sn, each overlaid with a semitransparent image of the grain boundaries derived from EBSD, except for a vertical band lying to the left of center of each image. The composition appears uniform but with concentrations of O and Cu at some grain boundaries. (h) A compositional phase diagram derived from the EDS data and indicating the Ti:Ni:Sn elemental ratio across the surface to be strongly peaked at 1:1:1.

The internal microstructure and composition of the TiNiCu_0.1_Sn sample is illustrated in Figure 1, which summarizes several analysis techniques. Contrast in the SEM image (Figure 1a) is dominated by white features that relate to Ti oxides at some grain boundaries, and dark features relating to surface roughness (notably the dark crosses that were FIB-milled as fiducial marks). Figure 1b is a map of granularity derived from an EBSD analysis, where the colors relate to the orientation of the crystallographic surface normal. Grains range in size from ∼2 to 20 μm diameter and a majority of the grain boundaries appear rough and meandering, consistent with what are termed “general” grain boundaries elsewhere^20^ and which lack well-defined crystallographic orientations. One such grain boundary is indicated by the first arrow in Figure 1a and is evident in both the SEM image and the oxygen map (Figure 1d). There is a smaller proportion of more geometric, regular boundaries that we find (see below) to have a degree of crystallographic alignment: one of these is indicated by the second arrow in Figure 1a. It is difficult to discern in SEM contrast but appears as a straight boundary in Figure 1b and lacks any obvious features in the oxygen map (Figure 1d). Figure 1c–g show maps of the elemental distributions derived from EDS, which indicate good uniformity of the main alloying elements. Each map is overlaid with an indication of the grain boundaries derived from the EBSD map, which together suggest a tendency for O and Cu enhancement at grain boundaries. Peaks in the O map are often found to coincide with slightly enhanced Ti and depleted Ni signals. (Ni depletion is most readily observed within the vertical stripe where the grain boundary overlay has been omitted for clarity, but the Ti enhancement is more subtle to discern.) Some of the largest features in the Cu map coincide with peaks in the Sn map and depleted regions of Ti and Ni, consistent with the formation of a trace volume-fraction of Cu–Sn alloy, similar to that discussed in a related hHA study^21^ and linked to a mineralizing effect that promotes grain growth and chemical homogeneity. The brightest feature in the O map, lying at the bottom right of Figure 1d, coincides with a peak in the Cu map (Figure 1f) and suggests a very low volume-fraction of copper oxide impurity. We find, however, that most of the copper exists in alloy or elemental form rather than as an oxide (see below) and do not find copper oxides to be significant. Finally, Figure 1h summarizes the compositional analysis, with the elemental ratios measured at each pixel positioned within a Ti–Ni–Sn phase diagram. The global composition derived from EDS has been normalized to the ratio of elemental powders used in synthesis, and the main observation is that the data are dominated by a single feature at the centroid, which is a clear indication of the presence of the TiNiSn phase. The peak’s sharp appearance indicates a relatively narrow degree of compositional variation across the sample. For example, the formation of a full-Heusler TiNi_2_Sn phase would produce a second spot immediately above the centroid but is not observed here. Even the Cu–Sn alloy phase identified above is insignificant. This relatively homogeneous composition is important because it indicates that nanocharacterization of a small number of samples extracted from the center of grains should be representative of the bulk material. In contrast, we have previously described hHA samples with pronounced compositional inhomogeneity, both within and between grains.^22,23^

**Figure 2:**
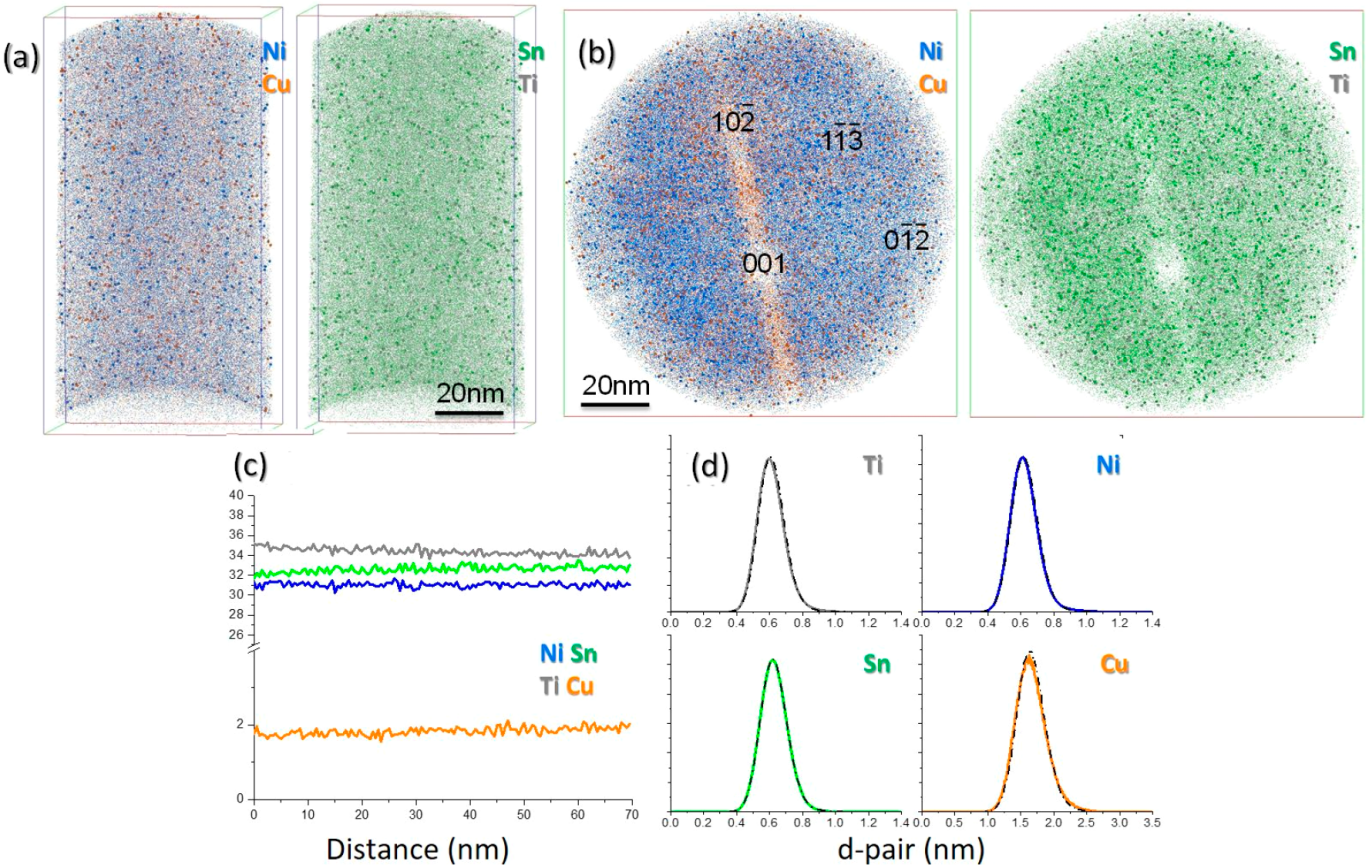
Atom-probe tomography analyses from bulk regions of TiNiCu_0.1_Sn. (a) Atom maps (side-on view) showing distribution of Ni + Cu and Sn + Ti atoms from a specimen extracted from the grain indicated by the white spot in Figure 1b. Data collected in laser-pulsed mode (100 pJ). (b) Atom maps (head-on view) showing distribution of Ni + Cu and Sn + Ti from a specimen extracted from a neighboring grain. Data collected in voltage-pulsed mode, with major crystallographic poles labeled. (c) One-dimensional composition profiles along the needle axis for the sample in (b), showing a uniform composition with no segregation or clustering apparent. (d) Nearest-neighbor analyses for Ti, Ni, Sn, and Cu from the sample shown in (b), where colored lines for each element overlap with dotted lines derived from a random distribution, indicating a lack of identifiable clustering. (*y*-Axes all scaled to (common) arbitrary units).

Figure 2 summarizes the results from two atom-probe needles extracted from two different grains, the first of which is indicated by the white spot in Figure 1b, representing the atom maps in Figure 2a. This was analyzed under laser mode (100 pJ). The data set reveals a uniform, homogeneous distribution of all elements with no evidence of phase segregation or clustering. The overall composition from this specimen was found to be Ti_35.1_–Ni_30.0_–Sn_32.6_–Cu_2.1_ (at.%), in reasonable agreement with the nominal chemistry (Ti_32.8_–Ni_32.8_–Sn_32.8_–Cu_1.6_)_._ The slightly high Ti/low Ni content is attributed to proportionally more Ni ions evaporating as partial hits, as documented previously,^19^ so that the actual composition is expected to have slightly more Ni and slightly less Ti than determined here. In our previous study,^19^ laser pulse mode was found to yield compositions that are closer to the known stoichiometry. This figure also shows the results from a specimen extracted from the center of a nearby grain and analyzed in voltage-pulsed mode. Figure 2b shows the resulting atom maps, in this case presenting data as if looking down the needle axis, revealing crystallographic poles deriving from the alloy’s fcc structure. (NB. The bands across the data set do not indicate regions of low density within the needle but arise from the crystallographic orientation and are known as zonelines.^24^) The microstructures in these atom maps again appear homogeneous, as does the resulting 1D composition profile in Figure 2c. The composition of this needle is determined to be Ti_34.5_–Ni_32.5_–Sn_31.3_–Cu_1.7_, close to both the nominal composition and that from the laser-pulsed mode specimen. The differences are ascribed to a combination of slight compositional variations between grains as well as minor differences in field evaporation behavior between the two analysis modes, as outlined previously.^19^ Finally, the plots in Figure 2d illustrate the outcomes from a nearest-neighbor analysis^25^ (*N* = 10) for all four species. Within each plot, the solid colored curves show the statistical separation of each M–M atomic pair, while the dotted curves are derived from the same atomic coordinates but with all ion identities now randomized. The real and randomized curves are coincident for all species, confirming homogeneous microstructures with no evidence of nanoscale clusters. Both the bulk composition and its uniform nature shown here also agree with findings of a preliminary study on this same material optimizing the atom-probe analysis conditions.^19^

An EELS analysis of the grain indicated by the white spot in Figure 1b yields a uniform composition of Ti: (32.5 ± 0.5)%, Ni: (37.0 ± 0.8)%, Sn: (30.5 ± 0.4)%, each expressed as the (mean ± standard deviation) atomic percent, determined from a spectrum image^26^ data set. Cu was undetectable to EELS because of the low doping level and because the Cu L_2,3_ EELS feature is broad and difficult to distinguish from the strong background in the EELS data set: this was one motivation for the APT analysis. The reason for the apparent enhancement of the Ni content measured by EELS is unclear and is being investigated separately.

The uniform composition is an important observation because segregation of full-Heusler or dopant-rich nanoparticles within the half-Heusler matrix has been used to introduce lattice-matched phonon scattering centers that reduce thermal conductivity without impairing electrical conductivity.^27,28^ Here, we find no evidence for either full-Heusler alloy inclusions or Cu-rich nanostructures within grains, in agreement with our previous diffraction studies,^13^ which suggests that at low concentrations, excess metal dopants are distributed randomly throughout vacant crystallographic sites in the hHA matrix. The formation of Cu-rich “full-Heusler” inclusions only became apparent in TiNiCu_*x*_Sn for Cu concentrations (*x*) above 0.1 (i.e., 3.2 at. %). Detecting such inclusions can be very difficult to find with other composition analysis techniques because the Cu dopant here is at a low percentage concentration and to detect inhomogeneities in the *distribution* of Cu requires high sensitivity to compositional fluctuations at near-atomic-scale, which is achievable using APT.

### Grain Boundary Analysis

**Figure 3:**
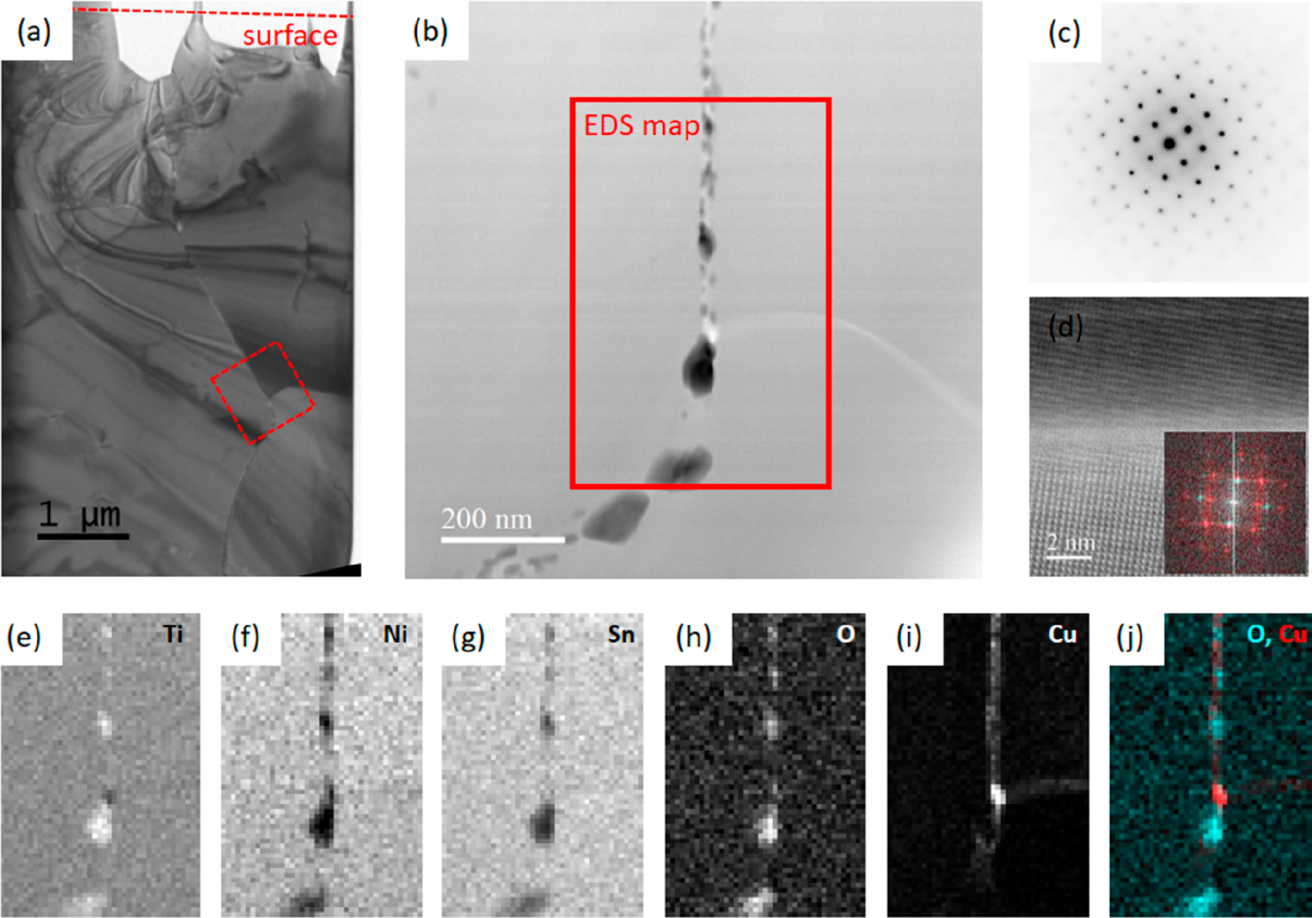
TEM/STEM analysis of the structure beneath the surface illustrated in Figure 1. (a) Low-magnification cross-sectional TEM image of a lamella extracted from a region close to the point indicated in Figure 1, with the surface plane indicated at the top. (b) STEM detail of the three-way grain boundary indicated in the TEM image. (c) Selected area electron diffraction pattern collected from the lower-right grain of image (b), consistent with scattering from a single crystal *F*4̅3*m* structure along the [001] direction. (d) High-resolution STEM image of the boundary between the two grains lying to the right of image (b) with (inset) overlaid Fourier transforms showing crystallographic alignment, red features deriving from the lower grain and cyan features from the upper grain. (e–i) Elemental maps derived from EDS analysis within the rectangular region indicated in (b). (j) Overlaid maps of the Cu (red) and O signals (cyan) collected from the same three-way grain boundary.

Figure 3 summarizes a TEM/STEM analysis of a ∼10 μm wide lamella extracted from a region close to that of the APT results. The low-magnification TEM image of Figure 3a indicates the structure beneath the surface of Figure 1 to have similar (and therefore isotropic) polycrystalline character to that of the surface. Contrast in the image is dominated by diffractive effects, and abrupt changes in the curved bend-contour fringes indicate grain boundaries, where there is an abrupt change in crystal orientation. The red box indicates the location of a higher magnification analysis that is described in subsequent panels, lying ∼5 μm beneath the surface in Figure 1 and straddling the boundary between three grains. Figure 3b is a dark-field STEM image of the threefold GB, where the brightest features correspond to the heaviest elements and materials. It indicates two main GBs: one running vertically through the whole image and characterized by a string of dark regions; and another GB running roughly horizontally on the right of the image, characterized by a narrow, lighter band. These two GBs are of the same two types as those identified in Figure 1. Figure 3c is an electron diffraction pattern that was collected from the lower-right grain of Figure 3a and which is typical of the grains running throughout the sample. It is consistent with diffraction from the *F*4̅3*m* structure, viewed along a [001] direction.

Figure 3d is a high-magnification STEM image of a region of the GB between the two grains lying to the right of Figure 3a, straddling the light band observed in dark-field STEM. The atomic lattice is evident in both upper and lower portions of the image. The inset shows fast Fourier transforms of top (cyan) and bottom (red) grains—the sharp spots, similar to diffraction patterns, are clearly aligned, indicating crystallographic registry of the two grains: the image is consistent with a typical low-angle grain boundary. No such registry was evident across the rough GB.

Figure 3e–j summarizes EDS analysis within the region indicated by the red box in Figure 3b. Ti, Ni, and Sn appear uniform in all three grains, except for the rough GB region, indicating the internal grain composition to be relatively homogeneous and without obvious segregation or nanostructuring effects. These images are dominated, however, by the rough GB features, which are rich in Ti and O (Figure 3e,h). The dark features in Figure 3b are therefore identified as titanium oxides. The Cu map, Figure 3i, shows a low Cu signal throughout the image, consistent with uniform dissolution within the grains themselves. It also indicates a thin band of Cu enrichment at both types of GBs, which accounts for their brighter appearance in dark-field STEM. For the smooth GB, the segregation of Cu and observation of coherent lattices on either side are consistent with the formation of Cu “wetting layers” that we have described previously and are attributed to extrusion of Cu from the bulk matrix during hot-pressing.^13^ These GBs are important because their structure suggests that they will have little impact on electronic conduction, but their structure could scatter long-wavelength phonons and thereby impair thermal conductivity. We will not discuss them further here. The segregation of Cu at the rough GBs is also interesting: segregation is, presumably, driven by the thermodynamics of limited solubility of Cu in the bulk hHA matrix—and the extrusion of impurities to grain boundaries has been observed in many other polycrystalline systems.^20^ Our previous diffractive studies of Cu-doped TiNiSn indicated a contraction of the bulk lattice parameter upon hot-pressing the samples during synthesis, which is consistent with the extrusion or dissolution of Cu from the bulk matrix.^13,29^Figure 3j, which is a false-color superposition of the O (in cyan) and Cu (in red) maps and clearly indicates that although both Ti oxides and Cu decorate this GB type, they are not completely coincident. There appears to be a low level of Cu throughout the GB and evidence for occasional Cu-rich precipitates (e.g., the red spot at the center of the three-way GB).

Resolving the structure of the precipitates at rough GBs, both copper and titanium oxides, is challenging in transmission electron microscopy because the images collected are the two-dimensional projections of these inherently 3D structures and because the spectroscopic signals from Cu, in either EELS or EDS, are weak and hard to distinguish from the background. We therefore turned to APT to discern the 3D structure of these features.

**Figure 4:**
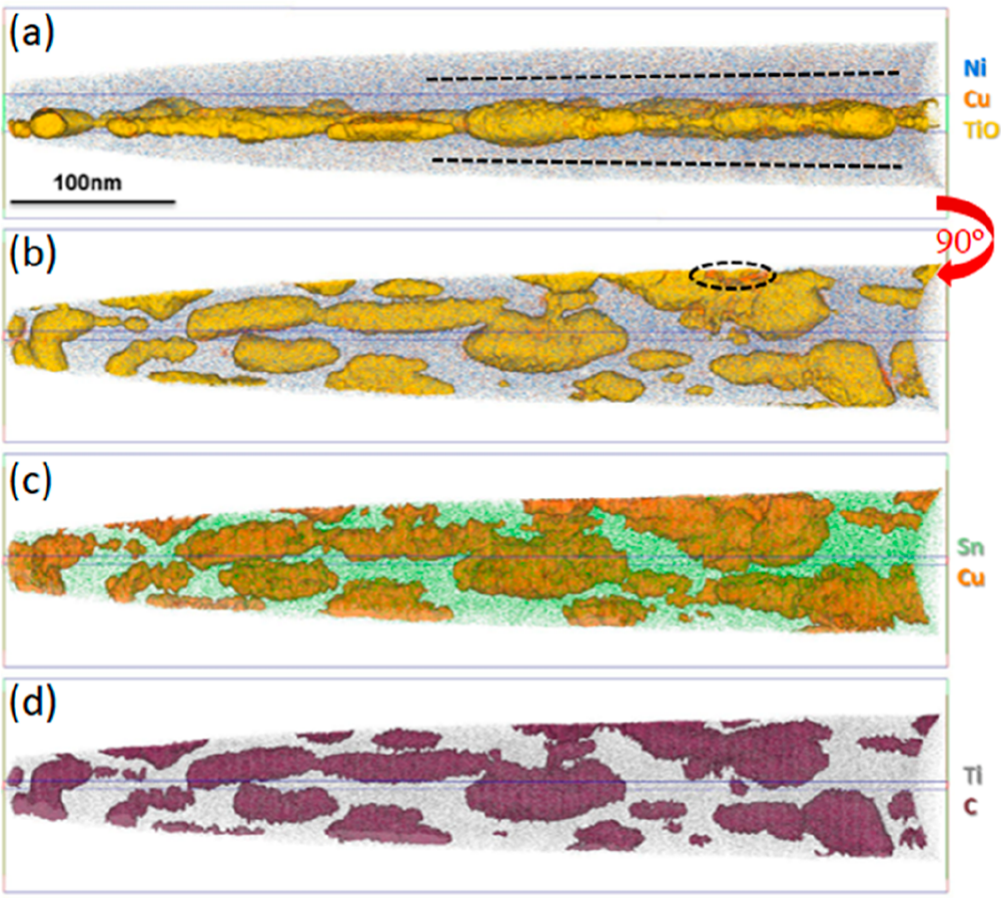
Atom maps highlighting detected species within an APT needle containing a grain boundary. (a,b) Ni and Cu distributions (blue and orange points) with a TiO isosurface in yellow. The map in (a) is rotated 90° relative to (b) and subsequent panels in order to highlight the flat grain boundary structure. (c,d) The same volume showing maps of (c) Sn and a Cu isosurface (in green and orange, respectively) and (d) the Ti and C distributions (in gray and maroon). The grain boundary is decorated by nanoscale precipitates, all of which appear enriched in TiO_*x*_, Cu, and C, as clearly shown by the respective isoconcentration surfaces (2% TiO, 3.5% Cu, and 0.6% C). For explanation of dotted lines in (a), refer to the main text.

Figure 4 presents APT atom maps (collected under laser pulse mode) from a ∼6 μm long needle with a rough GB running along its axis. Analysis of two subvolumes (cylindrical, 300 × 15 nm, centered on the dashed lines in Figure 4a and avoiding the oxide particles) yields a composition of Ti_35.2_–Ni_29.3_–Sn_33.0_–Cu_1.6_ for the upper grain and with the lower returning Ti_35.4_–Ni_28.5_–Sn_33.6_–Cu_1.8_. With the numerical counting errors on each typically ±0.05% (±0.01% in the case of Cu), the data are hinting at possible, slight variations in composition between grains, as commented above.

Figure 4a,b shows the same data viewed (a) along the GB and (b) perpendicular to the GB. The Ni distribution is indicated in blue, Cu is indicated in orange, and Ti oxides are indicated by the yellow isosurfaces that encapsulate volumes above 2 at. % TiO. From these images, it is apparent that the grain boundary is decorated with a number of discrete nanoscale clusters rich in Ti and O that correlate well with the titanium oxides revealed in projection by STEM. These vary in size from ∼10 up to ≥100 nm and present as both discrete and interconnected regions that are flattened within the grain boundary plane. Determining the precise stoichiometry of oxides can be complicated in APT by the production of neutral oxygen species that are not detected,^30^ and we therefore refer to the presence of TiO_*x*_ although consider the measured stoichiometry to be too rich in Ti to be consistent with TiO_2_, even accounting for underdetection of oxygen. In addition to TiO_*x*_ species, the precipitates are also rich in Cu (Figure 4c shows the 3.5% isosurface) and C (Figure 4d shows the 0.6% isosurface), as shown by the respective isoconcentration surfaces of these in Figure 4c,d. The Cu distribution is more obvious when viewed without the TiO isosurfaces, and the high spatial correlation between Cu, C, and TiO_*x*_ inclusions in all of these maps clearly suggest a degree of cosegregation. The dashed ellipse in Figure 4b indicates a region where the Cu lies on the outside of the Ti oxide, and this spatial relationship between elemental distributions is explored in more detail below. Oxygen is a common trace impurity in TiNiSn hHAs^21^ and is attributed to oxidation of the elemental powders used for synthesis and/or oxidation of the alloy surface during the first annealing stage of synthesis. Carbon is assumed to derive from impurities in the starting materials.

**Figure 5:**
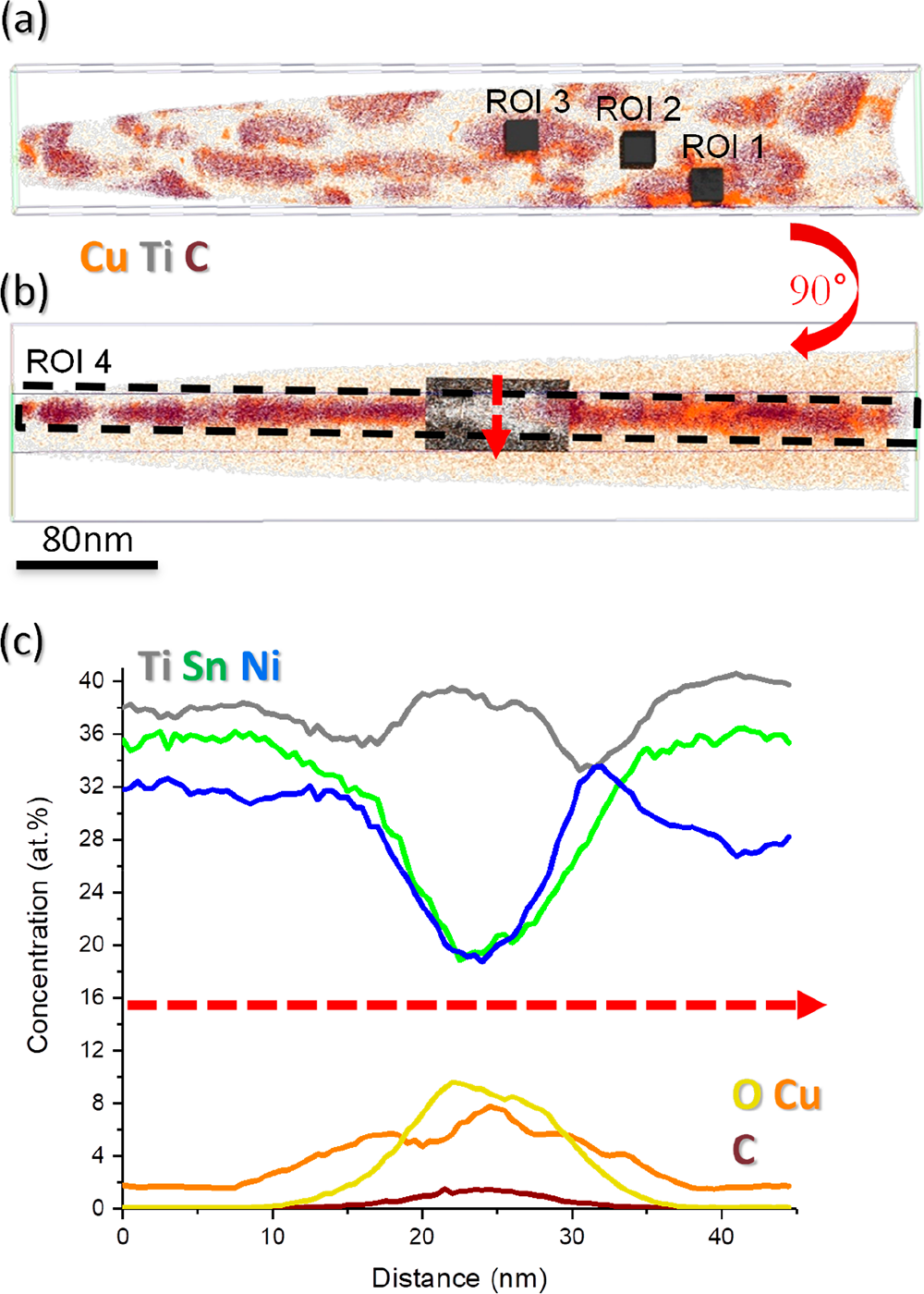
(a) Atom maps through the GB illustrated in Figure 4, showing the distributions of Cu, Ti, and C. Regions of interest (ROIs) are indicated and used to determine localized concentrations through the TiO inclusions (1,3) and in surrounding metal (2). (b) The same data, viewed at 90° to that of panel (a), in the plane of the GB. The dashed box demarks the ROI (4) used to define the whole GB volume. (c) Composition profiles through the black and white box indicated in panel (b), straddling the GB.

Figure 5 focuses on distinct regions of interest (ROIs) within the needle of Figure 4. Three ROIs (20 × 20 × 26 nm) cut across the grain boundary to investigate volumes containing the TiO_*x*_ inclusions (ROIs 1 and 3) and the alloy region (ROI 2). A fourth ROI, indicated by the dotted box (25 × 45 × 550 nm) in Figure 5b, captures all inclusions and neighboring matrix atoms to assess the overall GB chemistry.

The compositions of all four regions are shown in Table 1, which indicates that the whole GB (ROI 4) contains, on average, ∼5% Cu, around 3 times that of the bulk grains on either side of the GB. Within localized regions in close proximity to the TiO_*x*_ inclusions (ROIs 1 and 3), the Cu content is significantly higher, approaching 20 at. %, consistent with the proposed extrusion of Cu to the GBs during hot-pressing and the clear segregation observed in STEM. In contrast, in the hHA regions located between the inclusions (ROI 2), there is a depletion of Cu (1.4 at. %) compared to the bulk value; indeed, three additional ROIs placed similarly between the inclusions (not shown) all returned Cu contents of around 1 at. %. This degree of nanometer-scale, 3D chemical inhomogeneity would be very difficult to localize using STEM techniques alone.

**Table 1:** Composition (at. %) of the Grains Indicated in Figure 5, the ROIs Indicated in Figure 6, and the Oxide Inclusions Discussed in the Main Text^a^

Region	Ti	Ni	Sn	Cu	O	C
Upper grain	34.1	30.2	32.6	2.0	-	-
Lower grain	34.4	30.6	31.7	1.7	-	-
ROI 1 (inclusions)	34.7	13.5	12.4	19.9	14.1	3.0
ROI 2 (GB alloy)	34.1	31.3	32.2	1.5	0.3	0.2
ROI 3 (inclusions)	29.5	22.4	26.6	15.7	4.0	0.9
ROI 4 (whole GB)	34.3	27.9	29.0	5.46	2.0	0.7
Oxide inclusions	45.2	10.3	10.5	5.1	23.8	2.8

a The regions of interest (ROIs) select volumes that are predominantly oxide inclusions (ROI1 & ROI3), the alloy between the inclusions (ROI2), or the whole grain boundary, including both alloy and oxide inclusions.

Figure 5c shows a 1D composition profile through the GB, derived from the region highlighted in black/white in Figure 5b. Segregation of O and Cu at the GB is clear, alongside trace quantities of C that are attributed to impurities in the starting materials segregating out from the bulk. Although colocated with O, the Cu distribution is broader and therefore is inconsistent with the Cu existing as an oxide, which is discussed in more detail below. Ni and Sn contents both diminish at the GB, in agreement with the EDS images of Figure 4, each dropping to ∼20% at.%, while the enhancement of titanium arises from the presence of titanium oxides. Very similar segregation effects, with enhanced Ti and O concentrations and depleted Ni and Sn, were recently observed in thin film studies of TiNiSn, where we speculated that trace oxygen drew Ti out of the hHA matrix during deposition.^21^

**Figure 6:**
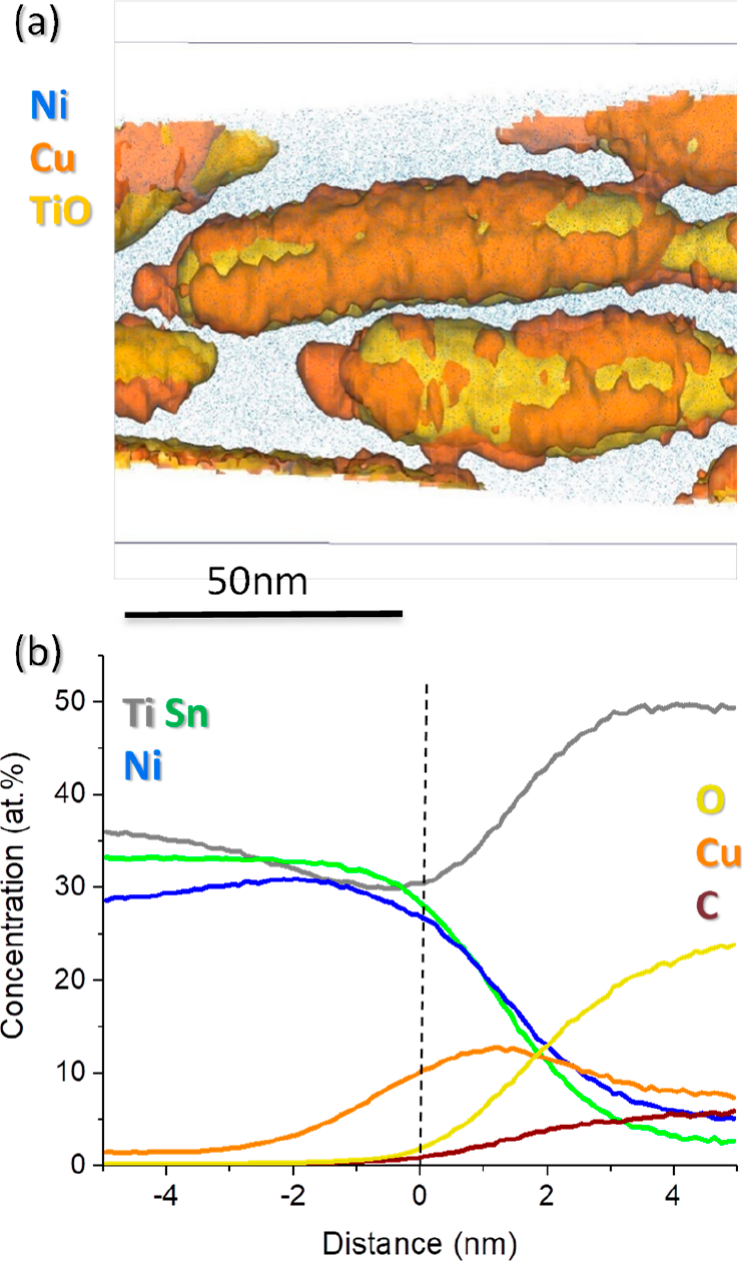
(a) Detail of the atom map of Figure 5, showing TiO (2 at. %) and Cu (3.5 at. %) isosurfaces in yellow and orange. Cu clearly decorates the perimeter of TiO-rich inclusions. (b) Proximity histogram (averaged from all TiO inclusions) confirms Cu-segregation peaks at the interface between the alloy matrix (left of vertical dotted line) and TiO-rich inclusions (right of dotted line).

Finally, we return to the relationship between Cu and Ti oxides at the GB. Figure 6 shows isosurfaces for TiO_*x*_ (2 at. %) and Cu (3.5 at. %) in a small section of the GB to highlight the matrix–inclusions interface. While the exact choice of isosurface values is somewhat open to interpretation, for all reasonable values for both TiO_*x*_ and Cu, Cu is located slightly outwith the TiO_*x*_, clearly indicating clustering of Cu to the interface of the titanium oxide particles. This clustering explains the broader Cu profile shown in Figure 5c and also the distributions of O and Cu illustrated in Figure 3j. It indicates the Cu to be present in metallic rather than oxide form, which was also the case for the Cu wetting layers observed in the more regular grain boundaries.^13^ The proximity histogram of Figure 6b shows aggregate composition profiles along the surface normal of the TiO_*x*_ inclusions, summed over all TiO_*x*_ inclusions. It clearly demonstrates that Cu is most concentrated at the interface between the oxygen-rich inclusions and the surrounding alloy matrix (approximate interface position as marked by vertical dotted line in Figure 6b). The TiO_*x*_ inclusions are also enriched in trace levels of C and N and therefore appear to have mopped up the main impurities from the bulk matrix. The overall composition of material within the TiO isosurfaces is indicated in the last row of Table 1. Nearly all of the oxygen is detected in APT as TiO^+^ molecular ions, with only trace levels (0.5%) of TiO_2_ present. There is approximately a 2:1 ratio of titanium detected as TiO vs Ti, as also apparent by eye in the proxigram, suggesting that the inclusions are either oxygen-rich regions or substoichiometric oxides that contain high Cu levels. Again, the presence of TiO_*x*_ inclusions rather than TiO_2_ was also observed in TiNiSn thin films;^21^ the present study may indicate that the oxides in the thin film case derived directly from the source material rather than forming in situ. Other oxides were not detected at significant levels. Although it would be expected that Ti oxides would form preferentially in a low-oxygen environment, the existence of elemental Cu and the lack of other notable oxides do suggest that the TiO_*x*_ inclusions and the Cu enrichment at grain boundaries arise from two distinct processes. For example, Cu extrusion may be expected to occur at high temperatures, while the segregation of already-formed TiO_*x*_ particles could occur earlier in the synthesis process. The resulting structures are not, for example, consistent with the diffusion of oxygen through the alloy to form oxides.

## Discussion

A previous study of TiNiCu_0.25_Sn and TiNiCu_0.1_Sn samples^13^ showed the GB chemistry to be enriched in Cu and focused on well-defined low-angle grain boundaries. Here, we have used a variety of techniques, spanning different length-scales, to characterize the chemistry of “rough” GBs first observed by SEM. The APT data presented here has shown that the GB in this TiNiCu_0.1_Sn material is again enriched in Cu but also in O, C, and N. The structure of rough GBs has a great deal of localized variation, with Cu segregating adjacent to oxygen-rich inclusions. These inclusions are substoichiometric oxides of titanium, containing 2–3 at. % of both C and N in addition to the elevated levels of Cu. Even within these there is further variation in the Cu distribution: on average around 5 at. % at the core but reaching 3–4 times this concentration at the interface between the matrix and titanium oxide inclusions. The morphology of the latter exist both as discrete regions and interconnected domains all lying along the plane of the grain boundary, between 10 and 100 nm in length. In contrast, in between the TiO-rich inclusions, the Cu content is actually depleted compared to the matrix, at around 1 at. % It is clear that these types of GB will impact both electronic and thermal transport and therefore impact on the performance of these materials as thermoelectrics. A main conclusion of this study is that better control over trace oxide formation is essential, and we note that to achieve this within an economic manufacturing process will be a challenge.

Segregation of impurities at GBs is well-known and has been observed in a number of materials systems, including other TEs.^31−33^ However, the inhomogeneous nature of the GBs described here is unusual. Extrusion of Cu to the grain boundaries during hot-pressing is of particular interest for a number of reasons. First, it suggests that the Cu acts as an efficient homogenizer and is mobile during synthesis, producing dense TE materials with largely homogeneous grain compositions and high-quality GBs that together improve electrical conductivity. Second, Cu extrusion would suggest a thermodynamic limit on the solubility of Cu within the TiNiSn hHA matrix around 2 at. %, which is surprisingly low. The half-Heusler alloys have long been seen as a remarkably tunable materials system, with vacant sites that can be readily doped to produce an alloy series ranging from XYZ to XY_2_Z, the “full”-Heusler. In practice, it is known that segregation into full- and half-Heusler alloys is favored over a uniform alloy series, but here, we have segregation in the form of the extrusion of excess metal rather than formation of FH inclusions. In contrast, we have shown that in the case of excess Ni and Co, segregation into full-Heusler domains will occur at similar dopant levels to those used here—the comparison is particularly relevant because of the similar atomic sizes and therefore of the lattice strain effects expected in each case.

Most intriguingly, extrusion of Cu to GBs in hHAs is similar to the liquid extrusion that has recently been used to boost the TE performance of Bi_2_Te_3_ and CoSb_3_ skutterudites: when molten Te (Sb) is extruded during pressure assisted consolidation, dense dislocation networks are observed to form at interfaces. Remarkably, these reduce the lattice component of thermal conductivity and improve electrical conductivity, leading to a 50% improvement in the thermoelectric performance (measured as “ZT”, a standard figure of merit for TEs^27^). The advance suggests it is possible to effectively decouple electrical and thermal scattering at GBs, and this type of GB engineering now needs to be explored in greater detail in the hHAs.

## Conclusions

We have demonstrated a multitechnique, multilength-scale approach to examine the micro and nanostructure of a TiNiCu_0.1_Sn thermoelectric material, utilizing SEM, EDS, EBSD, TEM, STEM, EELS, and APT methods. In this alloy, two main grain boundary types have been identified: GBs that appear rough in SEM imaging and are found to be decorated by discrete oxide precipitates and elemental impurities and more geometric GBs of well-defined crystallographic character. Copper is found to segregate at both GB types and has the potential to enhance thermoelectric performance. The nanoscale inclusions observed at rough GBs are a substoichiometric titanium oxide that capture excess Cu in addition to C and N contaminants. While copper colocates on these inclusions, it appears to concentrate at the matrix–inclusion interface and is furthermore depleted from the bulk value at purely metallic intersections between the two grains. The oxide formation at rough GBs will diminish electric transport through the bulk material and is expected to compromise thermoelectric performance. These findings suggest that routes to control the structure and chemistry of grain boundaries are essential to optimize the performance of future thermoelectrics.
